# Agglutinating Activity and Structural Characterization of Scalarin, the Major Egg Protein of the Snail *Pomacea scalaris* (d’Orbigny, 1832)

**DOI:** 10.1371/journal.pone.0050115

**Published:** 2012-11-20

**Authors:** Santiago Ituarte, Marcos Sebastián Dreon, Marcelo Ceolin, Horacio Heras

**Affiliations:** 1 Instituto de Investigaciones Bioquímicas de La Plata (INIBIOLP), CONICET CCT La Plata - Universidad Nacional de La Plata (UNLP), La Plata, Argentina; 2 Cátedra de Bioquímica y Biología Molecular, Fac. de Cs. Médicas - Universidad Nacional de La Plata (UNLP), La Plata, Argentina; 3 Instituto de Investigaciones Físicas Teóricas y Aplicadas (INIFTA), CONICET CCT La Plata - Universidad Nacional de La Plata (UNLP), La Plata, Argentina; 4 Cátedra de Química Biológica, Fac. de Cs. Naturales y Museo - Universidad Nacional de La Plata (UNLP), La Plata, Argentina; Consejo Superior de Investigaciones Cientificas, Spain

## Abstract

Apple snail perivitellins are emerging as ecologically important reproductive proteins. To elucidate if the protective functions of the egg proteins of *Pomacea canaliculata* (Caenogastropoda, Ampullariidae), involved in embryo defenses, are present in other *Pomacea* species we studied scalarin (PsSC), the major perivitellin of *Pomacea scalaris.* Using small angle X-ray scattering, fluorescence and absorption spectroscopy and biochemical methods, we analyzed PsSC structural stability, agglutinating activity, sugar specificity and protease resistance. PsSC aggluttinated rabbit, and, to a lesser extent, human B and A erythrocytes independently of divalent metals Ca^2+^ and Mg^2+^ were strongly inhibited by galactosamine and glucosamine. The protein was structurally stable between pH 2.0 to 10.0, though agglutination occurred only between pH 4.0 to 8.0 (maximum activity at pH 7.0). The agglutinating activity was conserved up to 60°C and completely lost above 80°C, in agreement with the structural thermal stability of the protein (up to 60°C). PsSC was able to withstand *in vitro* gastrointestinal digestion, and showed no trypsin inhibition activity. The presence of lectin activity has been reported in eggs of other *Pomacea* snails, but here we link for the first time, this activity to an apple snail multifunctional perivitellin. This novel role for a snail egg storage protein is different from closely related *P.canaliculata* defensive proteins.

## Introduction

Egg proteins of *Pomacea* are of particular interest since their functions seem to be related to the peculiar developmental environment of the embryos [1]. These freshwater snails have an unusual mode of oviposition as they lay their eggs outside the water [2]; [3]; [4], where they remain exposed to many environmental stressors like sunlight, desiccation and terrestrial predators during development [5]. Studies on *Pomacea canaliculata* eggs showed that their major perivitellins, ovorubin (PcOvo) and PcPV2, play important roles in coping with these stressors [6]. Both perivitellins are structurally stable at high temperatures, being PcOvo also stable at extreme pH values [7]; [8]; [9]. PcOvo is involved in the antioxidant system of the eggs, stabilizing and transporting a potent antioxidant from the perivitellin fluid to the embryo [10]. Also, both proteins would take part in egg defenses against predation; PcPV2 displays a neurotoxic effect in mice [11], and PcOvo is able to decrease rat growth rate when orally administrated, a novel antinutritive defense of the eggs [12]. PcOvo also provides the conspiuous reddish coloration to egg clutches, presumably a warning signal [6]. More recently, the major perivitellin of *Pomacea scalaris* eggs, scalarin (hereafter PsSC), was studied. This protein is a 380 KDa oligomer composed by three subunits of 35, 28 and 24 KDa, noncovalently bound. PsSC is also a carotenoprotein, with astaxanthin as a prosthetic group and, as PcOvo, it would be involved in the antioxidant system of the eggs [13]. PsSC shares structural features with PcOvo such as apoprotein composition, a high degree of glycosylation, and its prosthetic group [14]; however, the role of PsSC in embryo defenses was unknown.

Besides the novel defensive functions described for the perivitellins of *P. canaliculata*, a classical protective function reported for some snail egg proteins is lectin activity. Many lectins were isolated from gastropod eggs and organs which synthesize egg-coats, where they would be part of the innate immunity system of the snail protecting the eggs from bacterial invasions [15]. Among them, *Helix pomatia* Agglutinin (HPA), is undoubtedly the most studied gastropod lectin; it was isolated from the albumen gland and the egg perivitelline fluid and was thoroughly characterized both functionally and sructurally [16]. HPA is currently a valuable diagnostic tool in the biomedical field in histochemical studies relating glycosylation changes to the metastatic potential of solid tumors [17]; [18]. In the pulmonate *Biomphalaria glabrata*, two lectins belonging to C-type lectin family were isolated from the egg masses and for both proteins a protective role during the offpring development was suggested [19]. There are also reports on the presence of lectins in *Pomacea* snails, such as a thermally and pH stable lectin isolated from whole body extracts of *Pomacea flagellata* [20]; [21]. Besides, Ulenbruck and collaborators [22] reported the presence of agglutinins in the eggs of the ampullariid *Pila ovata* and Baldo and Uhlenbruck [23] described agglutinating activities in *Pomacea urceus* eggs and *Pomacea paludosa* albumen gland extracts, showing that both activities were inhibited by D-galactose, D-galactose derivates and oligosaccharides containing D-galatose.

In an attempt to determine if the protective functions reported in perivitellins of other species of *Pomacea* are present in *P. scalaris* perivitellins, in the present paper we study the trypsin inhibition capacity, agglutinating activity, and sugar specificity as well as the structural stability against temperature and pH of PsSC.

## Materials and Methods

### Ethics Statement

All the studies performed with goat, rat, horse, and rabbit erythrocytes were approved by the Institutional Review Board of the INIBIOLP and were carried out in accordance with the Guide for the Care and Use of Laboratory Animals [24]; (Instituto de Investigaciones Bioquimicas de La Plata's Animal Welfare Assurance No. A5647-01).

Human red blood cells were obtained from the Institute of Hemathology of the Province of Buenos Aires which is the government agency from the Ministry of Health that regulates the Provincial Hemotherapy System, framed within the Argentine National Blood Law No. 22.990/83 and the Provincial Act No. 11.725/95.

### Protein Purification

PsSC was purified from just-layed *P. scalaris* egg clutches by ultracentrifugation and size exclusion chromatography as previously described [13]. Protein concentration was measured by the method of Lowry [25]. Native and dissociating PAGE were performed in a Mini-Protean III System (Bio Rad, Hercules, CA) following manufacturer instructions, using molecular weight standards (GE Healthcare, Uppsala, Sweden).

### N-Terminal Sequence

Purified PsSC was sequenced by Edman degradation at the Laboratorio Nacional de Investigación y Servicios en Péptidos y Proteínas (LANAIS-PRO, Universidad de Buenos Aires–CONICET). The system used was an Applied Biosystems 477a Protein/Peptide Sequencer interfaced with an HPLC 120 for one-line phenylthiohydantoin amino acid analysis.

### Hemagglutinating Activity

Erythrocytes of goat, rat, horse, and rabbit were obtained from the animal facilities at University of La Plata (UNLP). In all species, blood samples were obtained by venous puncture and collected in sterile Elsever's solution (100 mM glucose, 20 mM NaCl, and 30 mM sodium citrate, pH 7.2) (Sigma-Aldrich, St. Louis, MO, USA). Human erythrocytes from healthy donors were obtained at the Institute of Hematology (Ministerio de Salud, Provincia de Buenos Aires, Argentina). Prior to use, erythrocytes were washed by centrifugation at 1500×g for 10 min in TBS buffer (20 mM Tris, 150 mM NaCl, pH = 7.4). This procedure was repeated several times until the supernatant remained clear. Hemagglutinating activity was assayed in microtiter U plates (Greiner Bio One, Germany) by incubating a two-fold serial dilution of PsSC (120 µg/ml) in TBS with 2% erythrocyte suspension in TBS at 37°C for 2 h. Results were expressed as the inverse of the last dilution showing visible hemagglutinating activity at naked eye.

### Sugar Specificity

PsSC sugar specificity was determined by comparing the inhibitory activity of the sugars galactose (Gal), glucose (Glc), mannose (Man), galactosamine (GalNH_2_), glucosamine (GlcNH_2_), N-acetyl-D-galactosamine (GalNAc), N-acetyl-D-glucosamine (GlcNAc) and the disaccharide sucrose, on the hemagglutination induced by the protein against rabbit erythrocytes. The inhibitory activity of the glycoproteins fetuin and transferrin was also assayed. For these assays, PsSC was two-fold diluted in a 200 mM solution of each sugar or a 0.5 mg/ml solution of the glycoproteins in TBS and incubated at 37°C for 1 h before adding the 2% rabbit erythrocytes suspension in TBS. The inhibitory capacity was expressed as the percent of inhibition, compared with the agglutination titer of PsSC without inhibitors: Inh% = [100− (Ti 100)/T], where T represents the hemagglutinating titer of the lectin without inhibitors and Ti titer with inhibitors.

### Effects of Metal Ions, Temperature and pH on Hemagglutinating Activity

The effect of divalent metal ions on the hemagglutinating activity of PsSC was assessed by testing the hemagglutinating activity of a PsSC solution (120 µg/mL) in the presence and absence of divalent metals (TBS supplemented with 10 mM MgCl_2_ or CaCl_2_). To study the effects of temperature on hemagglutinating activity, PsSC solutions (120 µg/mL) were incubated in a water bath at temperatures of 25, 40, 60 and 80°C for 1 h. The PsSC solutions were then immediately cooled on ice before the hemagglutinating activity assay. The effect of pH on the hemagglutinating activity was studied using PsSC solutions dialyzed for 24 h against buffer solutions at pH 2.0, 4.0, 6.0, 8.0, 9.0 and 12.0 prepared using phosphate and citrate sodium salts [26]. The hemagglutinating activity was then tested as described above.

### Trypsin Inhibition Assay

To test trypsin inhibition capacity of PsSC, a 0.5 mg/ml protein solution was incubated with a 10 fold molar excess of trypsin for 1 h, and trypsin inhibition determined following the method of Schwert and Takenaka [27]. In short, N-benzoil-L-arginine ethyl ester (BAEE) is hydrolyzed by trypsin at the ester linkage causing an increase in absorbance at 253 nm at 25°C.

### Effect of pH and Temperature on Structural Stability

To test the effect of pH on PsSC structural stability, purified protein samples were dissolved and incubated for 24 h in buffers at different pH values ranging from pH 2.0 to pH 12.0. Buffer formulations were taken from Merril [26]. After incubation, samples were analyzed by absorbance and fluorescence spectroscopy and by small angle X-ray scattering (SAXS) as described below.

The effect of temperature on scalarin stability was also measured in the range 25–85°C by absorbance and fluorescence spectroscopy and by SAXS.

### Absorbance and Fluorescence Spectroscopy

Sample absorbance was recorded between 300–600 nm in an Agilent 8453 UV/Vis diode array spectrophotometer (Agilent Technologies, Waldbronn, Germany). Three spectra were recorded and averaged for each sample and the corresponding buffer blank was subtracted.

Fluorescence emission was recorded in a Varian Cary Eclipse spectrofluorometer (Varian Inc., Australia). Emission was excited at 295 nm and recorded between 285–400 nm with a slit of 5 nm (both, in the excitation and emission path), in 5 mm optical path quartz cells, kept at 25°C (except for the thermal stability measurements). Three spectra were recorded and averaged for each sample and its corresponding buffer blank was subtracted.

### Small Angle X-ray Scattering

SAXS experiments were performed at the D02A-SAXS2 line operating in the Laboratório Nacional de Luz Síncrotron, Campinas (SP, Brazil). The scattering pattern was detected using a MARCCD bidimensional charge-coupled device assisted by Fit2d v12.012 software [28]. The experiments were performed using a wavelength of 1.448 Å for the incident X-ray beam to minimize carbon absorption. The distance between the sample and the detector was kept to 1044 mm, allowing a Q-range between 0.012 and 0.25 Å^−1^ (Dmax = 260 Å). The temperature of the cell was controlled using a circulating water bath, and kept to 25°C, (except for the thermal stability experiments). Each individual run was corrected for sample absorption, photon flux, buffer scattering, and detector homogeneity. At least three independent curves were averaged for each single experiment, and buffer blank scattering was substracted. To rule out a concentration effect in the data, SAXS experiments in a protein range of 3.0–0.2 mg/mL were performed.

### *In vitro* Gastrointestinal Digestion

Simulated gastric digestion of PsSC was performed at 37°C for 120 min at pH 2.5 in the presence of porcine pepsin (Sigma, Dorset, UK; product No. P 6887) at a ratio enzyme: substrate 1∶20 (w/w). Aliquots were taken at 0, 60 and 120 min and analyzed by SDS-PAGE as described above. The digestion was stopped by raising the incubation solution to pH 7.5 using 1.50 M Tris/HCl buffer. For *in vitro* duodenal digestion, the 120 min gastric digest was used as starting material. The simulated duodenal digestion was performed using trypsin from bovine pancreas (Sigma, product No. T 9935) at an enzyme: substrate ratio of 400: 1(w/w), at 37°C taking aliquotes at 0, 60 and 120 min for SDS-PAGE analysis. Albumin was used as positive (with enzyme) and negative (without enzyme) control in both gastric and duodenal digestion.

## Results

### Hemagglutinating Activity and Sugar Specificity of PsSC

PsSC showed hemagglutinating activity against several red blood cells, being highest against rabbit erythrocytes (1/5120) and agglutinating to a lesser extent human, mouse, goat and rat erythrocytes. Human erythrocytes reacted differently according to the blood group. While A group was less reactive than B group, 0 group was not reactive in the hemagglutination assays (Table 1). Different carbohydrates were tested for their ability to inhibit hemagglutination, among them, Gal-NH_2_ was the most powerful inhibitor of PsSC activity (99.99% inhibition at 200 mM), followed by Glc-NH_2_ (99.60% inhibition) and GalNAc (50.0% inhibition). On the other hand, Man, Glc, Gal, Fru, GluNAc and sucrose showed no inhibitory capacity (Table 2). In addition, the glycoproteins fetuin and transferrin were tested and showed no inhibitory effect (data not shown).

**Table 1:** Hemagglutinating activity of PsSC against different types of mammalian erythrocytes.

Erythrocyte Type	Hemagglutinating activity titre
Rabbit	5120
Goat	20
Rat	40
Horse	0
Human-A	320
Human-B	640
Human-O	0

Results are based on 6 determinations for each RBC type.

**Table 2:** Inhibition of PsSC agglutinating activity by carbohydrates and glycoproteins.

Carbohydrate	% Inhibition
GalNH2	99.9
GlcNH2	99.6
GalNAc	50.0
GlcNAc	0.0
Glucose	0.0
Galactose	0.0
Fructose	0.0
Sacarose	0.0
Transferrin	0.0
Fetuin	0.0

* Results are based on 6 determinations for each carbohydrate.

### Effect of Metal Ions, Temperature and pH on PsSC Hemagglutinating Activity

Hemagglutinating activity of PsSC against rabbit erythrocytes was not modified by the addition of 10 mM CaCl_2_ or MgCl_2_. Hemagglutination also remained unaltered after pre-incubation of the protein at temperatures ranging from 25°C to 60°C; at higher temperatures the activity was completely lost (data not shown). The effect of pH on hemagglutinating activity was analyzed preincubating PsSC in buffers ranging from pH 2.0 to 12.0. In these experiments, hemagglutinination was observed between pH 4.0 and 8.0, while at higher or lower pH values the activity was completely lost (Data not shown).

### Effect of pH on PsSC Structural Stability

The structural stability of PsSC at different pH values was evaluated by fluorescence spectroscopy, absorbance spectroscopy and SAXS.

**Figure 1:**
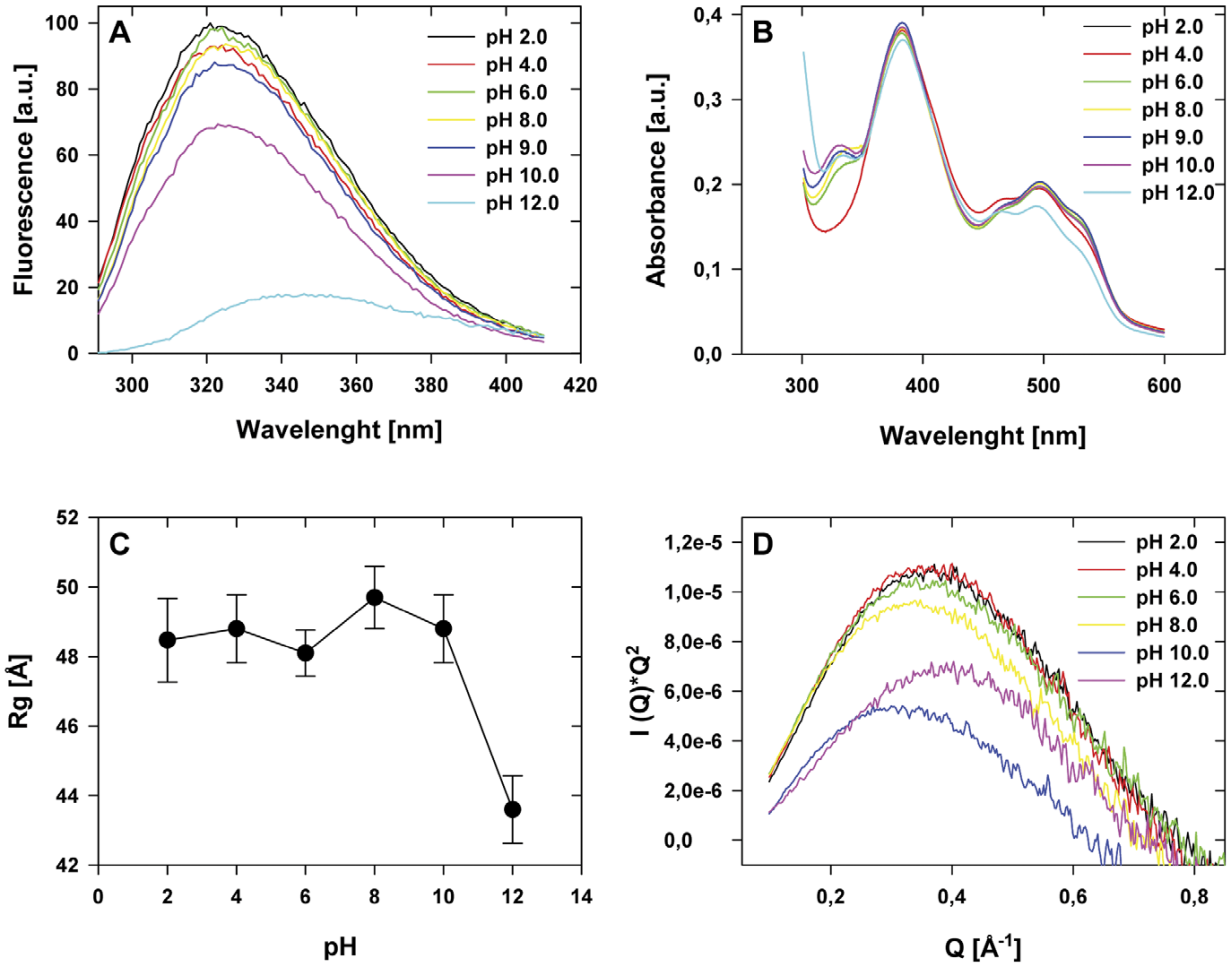
Effect of pH on PsSC structural stability. a) Tryptophan fluorescence emission spectra; b) Absorption spectra in the carotenoid fine structure region; c) Gyration radii (Rg) obtained from the SAXS data; d) “Kratky plot” (I(Q)*Q^2^ vs. Q) obtained from the SAXS data.

Tryptophan fluorescence emission spectra recorded between pH 2.0 and 9.0 was the expected for indole rings buried in hydrophobic environments, indicating that PsSC remains properly folded (Figure 1a). At pH 10.0 a partial quenching of the signal was observed, suggesting alteration of the tryptophan environment, more noticeable at pH 12.5 where a red-shift of the emission maxima was observed.

Absorption spectra in the region of the carotenoid cofactor absorption (430–550 nm) showed only minor fine structure alterations. Though changes in the 340 nm absortion band were observed at pH 4.0, only at pH 12.0 a decrease in intensity and a slight blue shift of the 494 nm band were evident, indicating slight perturbations in the hydrophobic environment of the pigment (Figure 1b). SAXS experiments showed that the gyration radius (Rg) of PsSC remains almost unchanged between pH 2.0 and 10.0. However, a sudden decrease at pH 12.0 can be observed (Figure 1c). Kratky plots (I(Q)*Q^2^ vs. Q) of the scattering data display a clear bell-shape, characteristic of well-defined globular structures, up to pH 12.0. However, the shift of the maximum to higher Q values indicates a decrease of particle size, consistent with the decrease of Rg (Figure 1d).

### Effect of Temperature on the Structural Stability of PsSC

**Figure 2:**
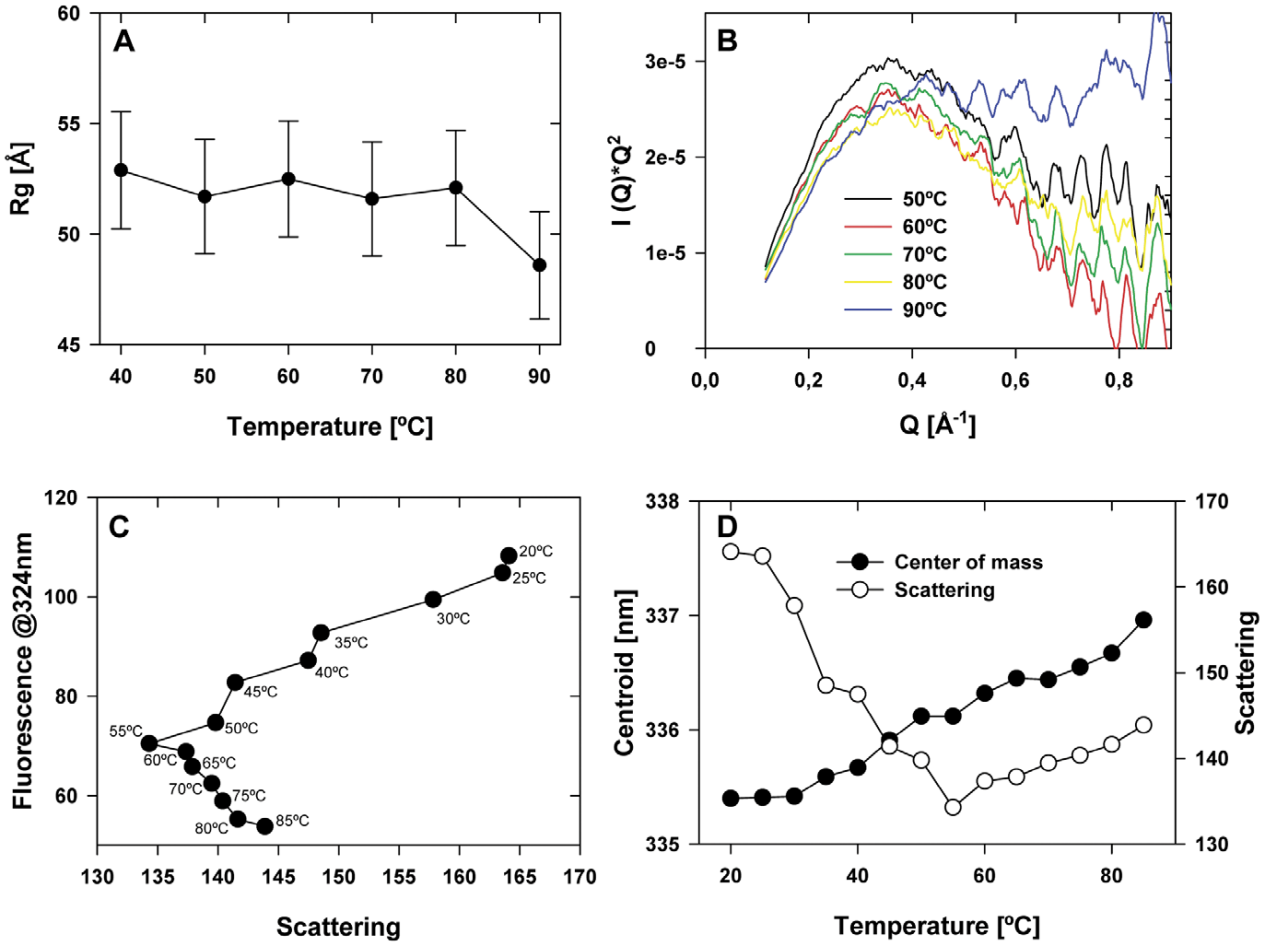
Effect of temperature on the structural stability of PsSC. a) Gyration radii (Rg) obtained from the SAXS data b) “Kratky plot” (I(Q)*Q^2^ vs. Q) obtained from the SAXS data. c) Phase diagram obtained from the tryptophan fluorescence data. d) Center of mass of the fluorescence spectra (solid circles) and elastic scattering intensity (I(295 nm), open circles) obtained from tryptophan fluorescence experiments.

The structural stability of PsSC at different temperatures was evaluated by SAXS and fluorescence spectroscopy. Figures 2a and b show SAXS data between 25°C–90°C, it can be seen that although the protein suffers alterations, there are no indications of complete unfolding. Analysis of the phase diagram [29]; [30] (figure 2c) obtained using the elastic scattering signal of the fluorescence spectra (I(295 nm)) and the tryptophan emission (I(324 nm)) indicates that the thermal evolution of PsSC is characterized by the presence of an intermediate state mostly populated around 55°C. The analysis of the center of mass of the fluorescence spectra (between 310 nm and 400 nm) (Figure 2d) shows that above 55°C the fluorescence intensity increases indicating a progressive exposure of the tryptophan residues to the solvent. However, up to 90°C the tryptophan residues were not completely exposed to a polar environment (λ_max_ = 337.5 nm) suggesting that even at high temperature the unfolding of the protein was not fully achieved [30]; [29].

### Simulated Gastrointestinal Digestion of PsSC

**Figure 3:**
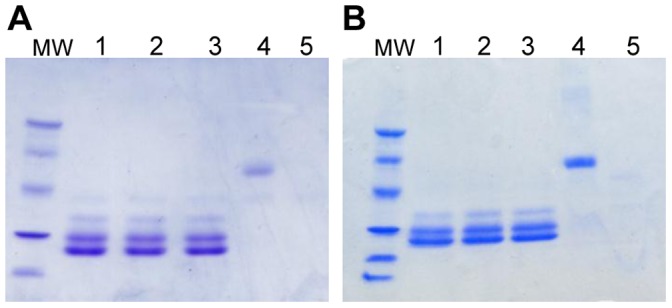
*In vitro* digestibility of PsSC analized by SDS-PAGE. A: Gastric digestion. Lanes 1–3: PsSC after incubation with pepsin for 0, 60 and 120 min; lanes 4 and 5: negative and positive control, respectively. B: Duodenal digestion. Lanes 1–3: PsSC exposed to trypsin for 0, 60 and 120 min, respectively; lane 4 and 5: negative and positive control respectively. Positive control: BSA with enzyme. Negative control: BSA without enzyme. MW: Molecular weight markers of 97, 66, 45, 30, 22.1 and 14.4 KDa.

Protease resistance was assayed in an *in vitro* gastrointestinal digestion experiment. Figure 3a shows the gastric phase (pepsin treatment at pH = 2.5) where only minor digestion can be observed after 2 h. The duodenal phase (trypsin treatment at pH = 8.0), indicates that PsSC resists incubation when exposed sequentially to both digestive proteases (Figure 3b).

### Trypsin Inhibition

In order to determine if PsSC shared with PcOvo trypsin inhibition activity, we performed a trypsin inhibition test. The protein was incubated with trypsin at molar ratios between 1∶1 and 10∶1 (PsSC: trypsin) and no inhibition of the protease activity could be observed (results not shown).

### N-Terminal Sequence

N-terminal amino acid sequence of deglycosilated PsSC was determined. A search against Swiss Prot database revealed no homology with any known sequence, though a high homology with N-terminal sequence of PcOvo was observed (Table 3).

**Table 3:** N-terminal sequence of PsSC, comparison with N-terminal sequence of PcOvo (from *P. canaliculata*).

	N-terminal sequence
PsSC	-DEXLLLDIIDASTEEIN
PcOvo^a^	NKEYLLLDIRDATTSEII
Conserved Residues	* ***** ** * **

a taken from Dreon *et al.*, [12].

## Discussion

### 1. Hemagglutinating Activity of PsSC

The major egg protein of *P. scalaris*, PsSC, shows strong agglutinating activity against rabbit erythrocytes, and a moderate activity against human A and B group erythrocytes. The presence of substances that agglutinate rabbit and human erythrocytes was reported in eggs extracts of other ampullariid snails namely *P. canaliculata* and *Pila ovata* and also in the albumen gland of *Pomacea urceus* [23]; [22].

Our inhibition experiments pointed out at Gal-NH_2_ and GlcNAc as powerful inhibitors of the hemagglutinating activity of PsSC, followed by GalNAc; it is of note that Gal did not inhibit hemagglutination (at 200 mM). A selective affinity for Gal and Gal-derived sugars was reported not only for lectins from other *Pomacea* snails (egg lectins from *P. urceus* and *P. flagellata*, [23] and adult body lectins from *P. flagellata* [20]), but also from other snails like *Achatina* [31]. As a whole, these results agree with previous studies suggesting that lectin specificity in molluscs is relatively conserved for galactosides [32].

The agglutinating activity of PsSC with human A and B groups is also in agreement with this conserved specificity for galactosides. The human blood group ABO antigens are oligosaccharides, part of glycoproteins and glycolipids, of the erythrocyte surface. The terminal residues of A and B blood group antigens are modifications of H-antigen carbohydrate (Fucα1-2Gal) and terminate in GalNAcα1-3(Fucα1-2)Gal and Galα1-3(Fucα1-2)Gal, respectively [33]. Thus the sugar specificity of PsSC is coincident with this terminal saccharide residues, suggesting these surface oligosaccharides are likely involved in the interaction between the lectin PsSC and the erytrhocytes.

The agglutinating activity of PsSC was rather pH stable (between pH 4.0 and pH 8.0) and independent of the presence of divalent metals. In this sense, *P. flagellata* whole-body agglutinins (PFA-I and PFA-II) present a similar behavior, although they retain activity up to pH 10 [20]. The effect of temperature on lectin biological activity also showed similarities between PsSC and PFA-I and PFA-II as hemagglutination activity was not affected up to 60°C in any of them.

### 2. Structural Stability of PsSC

The results presented here show that PsSC shares structural resistance towards extreme pH and temperature with *P. canaliculata* perivitellins. However, PsSC pH stability range includes much more acidic pH values (in fact, structural perturbations arise only at extreme alkaline values) while PcOvo is stable between pH 4.0 and 10.0 [8]. It is intriguing why PsSC agglutinating activity is lost between pH 8.0–10.0. Clearly, the loss of agglutinating activity is not due to major structural changes, as indicated by the unmodified SAXS signals. Moreover, the lack of activity is not associated to modifications in neither the local environment of the tryptophan residues, as reflected by the fluorescence spectra, nor in the carotenoid environment. It is more likely that the agglutinating activity is affected by alterations in the critical electrostatic interactions in lectin-carbohydrate recognition, not relevant to the stabilization of protein structure.

Thermal stability is another characteristic shared with *P. canaliculata* perivitellins, notably high in PcOvo (>95°C) [7] and moderate (up to 60°C) in PcPV2 [9]. In comparison, PsSC structure showed moderate to high thermal stability. Although partial unfolding is observed at temperatures higher than 55°C, at 90°C the protein still does not show signs of complete denaturation; agglutinating activity, however, was lost above 60°C. Gradual loss of activity above 60°C was also observed in *P. flagellata* agglutinins [20].

As the function of a protein depends on its structure, these common structural and stability characteristics of all *Pomacea* perivitellins analyzed so far may be a key acquisition to withstand the harsh out-of-the water environment as well as the digestive tract of potential predators [12].

### 3. Physiological Implications

The physiological explanation for the presence of a thermal and pH stable lectin inside the eggs of *P. scalaris* is far to be fully understood. Nevertheless, several possible explanations can be hypothesized. First, taking into account the role of lectins in the invertebrate immune system and the capacity of PsSC to specifically recognize oligosaccharides from cell surface, we could hypothesize a role of the protein in protecting the eggs from pathogen invasion.

Other potential roles that this perivitellin could have would be in embryo defenses against predation. The resistance of PsSC to gastrointestinal proteases together with its structural stability against pH could be interpreted as part of an antinutritive defense similar to that reported for PcOvo in *P. canaliculata* eggs, which would impair the acquisition of nutrients by a potential predator (antinutritive role). Moreover, pH stability would allow PsSC to reach the small intestine of its predators in an active form, as described for PcOvo, raising the hypothesis that both also shared the capacity to inhibit trypsin [12]. This possibility was reinforced by the high homology between N-terminal amino acid sequence of PsSC and PcOvo. The roles of these two perivitellins, however, are not equivalent as PsSC lacks the capacity to inhibit tripsin, discarding an antidigestive role in embryo defenses.

Finally, there is still another potential function of PsSC in egg defenses: if we consider its resistance to gastrointestinal digestion, together with its agglutinating activity, it may be possible that PsSC would be involved in egg defense through a mechanism not reported in animals, but similar to that described for some plant lectins. In this regard many studies have thoroughly established that lectins have a role in plant embryo defense against predation [34]. Further, it has been reported that plant seed lectins resistant to gastrointestinal digestion have deleterious effects on intestinal epithelium, interfering with the digestion and absorption of nutrients [35]. Further biochemical and physiological work is needed to elucidate the role that this lectin plays in the reproducitve strategy of the snail.

In conclusion, this report describes a novel function for a perivitellin and shows that even closely related species have evolved quite different embryo protective proteins, which adds to the recent interpretation of the multiple roles of perivitellins in the reproductive strategy of *Pomacea* snails [12].
